# Response after treatment with pembrolizumab in a patient with myelophthisis due to melanoma: the role of checkpoint inhibition in the bone

**DOI:** 10.1186/s40425-017-0236-3

**Published:** 2017-04-18

**Authors:** Samuel Rosner, Filiz Sen, Michael Postow

**Affiliations:** 1 Albert Einstein College of Medicine, 1300 Morris Park Ave, Bronx, NY 10461 USA; 2 Memorial Sloan Kettering Cancer Center, 1275 York Ave, New York, NY 10065 USA; 3 Weill Cornell Medical College, New York, USA

**Keywords:** Myelophthisis, Melanoma, Bone marrow, Immune checkpoint inhibition, Pembrolizumab, PD-1 inhibitor

## Abstract

**Background:**

Myelophthisis due to melanoma is a rare phenomenon. Treatment strategies for patients with this serious complication of malignancy have not been well documented, and none have previously reported efficacy of immune checkpoint inhibition. Since bone metastases are not measurable lesions per standard response criteria, the efficacy of immune checkpoint inhibition in the bones is also not well described.

**Case presentation:**

We describe a patient with widespread melanoma metastases involving the bone marrow causing myelophthisis and pancytopenia who responded to immune checkpoint inhibition with the anti-programmed cell death-1 (PD-1) inhibitor pembrolizumab.

**Conclusions:**

This is the first report to our knowledge of disease response to immune checkpoint inhibition in a patient with myelophthisis. Clinical trials have recently emerged describing the efficacy of PD-1 inhibition for disorders regularly involving the bone marrow, such as hematologic malignancies, suggesting the importance of better understanding the bone marrow as an immunologically active compartment. Clinicians should be aware that immune checkpoint inhibition alone may be effective in treating malignancy involving the bone marrow, even in cases of extensive involvement resulting in pancytopenia due to myelophthisis from a solid tumor as our case suggests.

## Background

Myelophthisis due to malignancy is a rare, but often significant, complication of cancer that occurs when metastases infiltrate bone marrow and impair normal hematopoiesis [1]. Although metastases from any solid tumor can lead to myelophthisis, descriptions of this phenomenon in patients with melanoma remain limited [2]. Due to its rarity, very little is known about treatment outcomes, and prior case reports have indicated only limited efficacy of chemotherapy [3–5]. Many new therapies are now available to treat metastatic melanoma such as immune checkpoint inhibitors, but whether immune checkpoint inhibitors are effective in patients with bone marrow infiltration and myelophthisis remains unknown.

We describe the first case to our knowledge of a patient with widespread melanoma metastases involving the bone marrow causing myelophthisis who responded to immune checkpoint inhibition with the anti-programmed cell death-1 (PD-1) inhibitor pembrolizumab.

## Case presentation

An otherwise healthy 64 year old male presented in 2/2015 with a suspicious lesion on his back. Biopsy and subsequent wide local excision revealed a 1.2 mm deep, non-ulcerated cutaneous melanoma. A sentinel lymph node biopsy involving his right groin in 4/2015 revealed one lymph node involved with micrometastatic (<1 mm) melanoma. According to the 7th edition of the American Joint Committee on Cancer (AJCC) staging system, he had stage IIIA melanoma (pT2AN1A). A computerized tomography (CT) scan in 5/2015 was unremarkable for metastatic disease, and he elected to not proceed with a completion lymph node dissection. The patient considered adjuvant systemic treatment options, and he chose to proceed with radiographic surveillance alone. Basic laboratory testing including a complete blood count (white blood cell count, hemoglobin, and platelets) was normal.

**Fig. 1:**
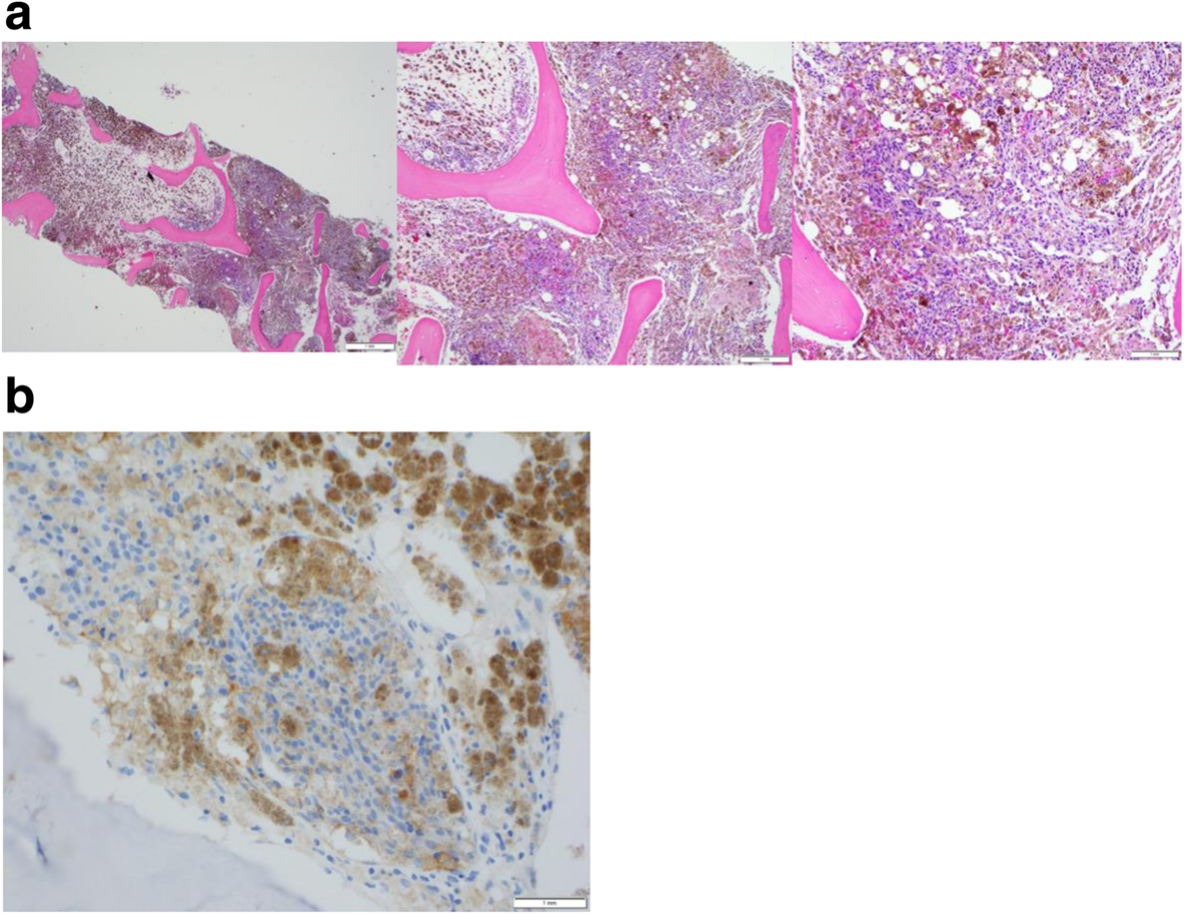
Image of patient’s bone marrow biopsy, confirming metastatic melanoma to bone with corresponding PD-L1 staining. Panel **a** shows images of the patient’s bone marrow with extensive involvement by metastatic melanoma. Panel **b** shows corresponding bone marrow sample with tumor cells staining positive for PD-L1 (Vendor: Cell signal, Clone: E1L3N, Dilution: 1:500)

Three months later, in 8/2015, the patient developed flu-like symptoms and fatigue with an accompanying low grade fever. He presented to the emergency room where blood work revealed new pancytopenia with a low white blood cell count of 3.1 (K/mcL) with a normal differential (Neutrophils 58%, Lymphocytes 23.5%, Monocytes 4.2%, Eosinophils 2.5%, Basophils 0%) and no immature cells. Hemoglobin was slightly low at 12.3 (g/dL), and platelets were low at 67 (K/mcL). He had no evidence of abnormal bleeding or bruising. A CT scan on 8/19/15 showed multiple lesions in liver, spleen, and some small pulmonary nodules consistent with metastatic disease. A liver biopsy confirmed metastatic melanoma, BRAF wildtype. Due to his pancytopenia, a bone marrow biopsy was additionally performed. Hematoxylin and eosin staining revealed extensive metastatic melanoma with decreased number of myeloid, erythroid and megakaryocyte lineages along with prominent melanophages (Fig. 1a). The melanoma cells stained positive for S100, HMB45, and SOX10. Although immunohistochemical staining for the programmed cell death ligand 1 (PD-L1) protein on melanoma cells is not a standard test in melanoma, for research purposes to better characterize the immunologic aspects of the metastatic melanoma involving the bone marrow, immunohistochemical staining was performed. PD-L1 staining results using the antibody E1L3N (Vendor: Cell signal, Dilution: 1:500) were positive (Fig. 1b).

**Fig. 2:**
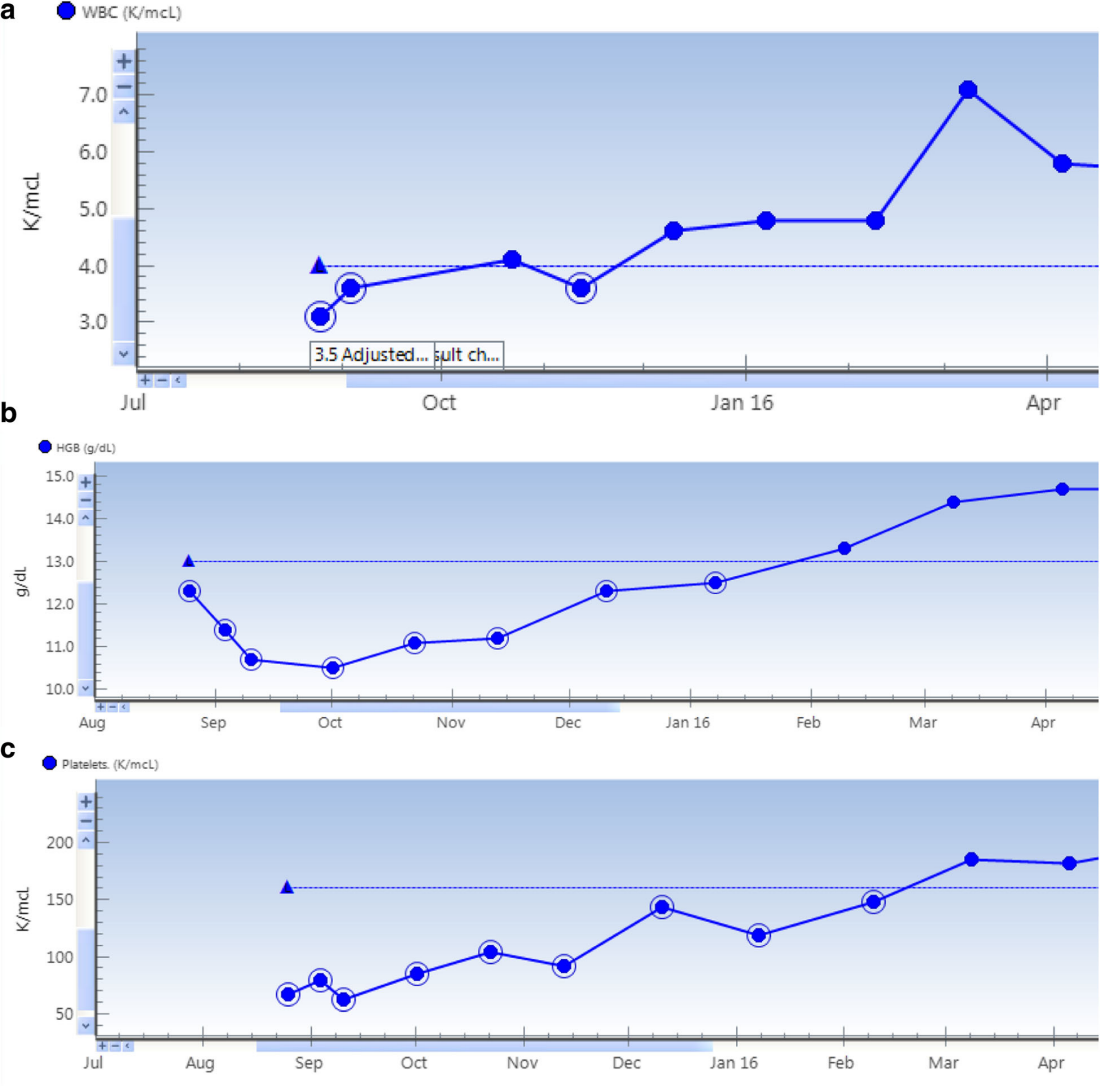
Change in patient blood counts over course of treatment with Pembrolizumab. Panel **a** indicates changes in patient’s white blood cell count (K/mcL) over the period of August 2015 through April 2016. Panel **b** indicates changes in the patient’s hemoglobin level (g/dL) over this same time period. Panel **c** corresponds to changes in the patient’s platelet level (K/mcL) over this same period

He started systemic therapy with pembrolizumab on 9/10/15. Within 3 weeks of starting pembrolizumab, he began to feel better with improvements in fatigue and functional status. Laboratory results from 12/10/15 showed improvements in his pancytopenia (Fig. 2), and a CT scan showed decreasing size of his liver lesions with persistent splenic lesions. By 7/2016, his splenic lesions had completely disappeared, and his hepatic lesions had continued to slowly shrink. He has remained on pembrolizumab and received a total of 19 treatments, which he has tolerated well aside from mild treatment-related vitiligo. His favorable outcome is ongoing, most recently documented on scans from 12/2016 showing stability of small liver lesions and no splenic lesions. His complete blood count remains normal.

## Discussion and Conclusions

Myelophthisisis occurs when advanced malignancy infiltrates bone marrow and impairs normal myelopoiesis. Presentations of myelophthisis can include anemia, thrombocytopenia, neutropenia and varying degrees of pancytopenia. Treatment is generally directed against the underlying malignancy with supportive transfusions as needed, but the condition can be refractory to standard treatments such as chemotherapy [3, 6].

With the emerging field of immune checkpoint blockade for cancer, little is known about these new agents and their efficacy in treating solid tumors involving the bone marrow or more generally, metastatic lesions present in the bones. Some of this void in information may be due in part to the utilization of the Response Evaluation Criteria in Solid Tumors (RECIST) response criteria for assessment of therapy effectiveness [7]. Since lesions present only in the bone without a soft tissue component are not considered measurable disease, the efficacy of immune checkpoint inhibition in the treatment of bone metastases remains limited as almost all pivotal studies of PD-1 immune checkpoint inhibitors have used RECIST criteria for response evaluation. In addition, clinical trial protocols commonly exclude patients with low blood cell counts, preventing the assessment of their efficacy in patient populations with bone marrow infiltration causing low blood counts.

The emergence of immune checkpoint blockade against hematologic malignancies which often involve the bone marrow, such as Hodgkin lymphoma, has provided a glimpse into the potential benefits of these therapies beyond their initially described uses for solid tumors. Recent results from the KEYNOTE-013 study reporting the use of pembrolizumab for patients with classical Hodgkin lymphoma showed an overall response rate of 65% with the majority of those responses (70%) lasting longer than 24 weeks, all in a cohort of patients who were heavily pretreated and refractory to multiple lines of therapy [8]. An additional Phase 1b study assessed the safety and efficacy of nivolumab for patients with non-Hodgkin lymphoma and multiple myeloma. Overall nivolumab was well tolerated and exhibited antitumor activity in extensively pretreated patients with various forms of relapsed or refractory B- and T-cell lymphomas [9]. Neither of these previously published studies, however, specifically reported whether immune checkpoint inhibition was effective in the bone marrow.

Our report of a profound melanoma response within the bone marrow to pembrolizumab suggests the bone marrow is an immunologically active compartment. Yet, whether the mechanisms involved in antitumor immune responses to checkpoint inhibition within the bone marrow are similar to those at other sites of disease remains unknown. Some literature suggests receptor activator of nuclear factor-Kappa-B ligand (RANKL), which can be produced by osteoblasts, may be involved in influencing dendritic cells to enhance T regulatory cell immune attenuation [10]. RANKL has inversely correlated with clinical outcomes, suggesting RANKL directed therapies such as denosumab may result in favorable antitumor immune effects [11]. Clinical trials, however, are needed to test whether the efficacy of immune checkpoint inhibition could be affected by combination therapy with a RANKL inhibitor. In the meantime, clinicians should be aware that immune checkpoint inhibition alone may be effective in the bone marrow, as our anecdotal case describes, even in cases of extensive involvement resulting in myelophthisis.

## Abbreviations

CT: Computerized tomography
PD-1: Programmed cell death-1
PD-L1: Programmed cell death ligand-1
RANKL: Receptor activator of nuclear factor kappa-B ligand
RECIST: Response evaluation criteria in solid tumors
